# Monoclonal Antibodies Specific for Disease-Associated Point-Mutants: Lamin A/C R453W and R482W

**DOI:** 10.1371/journal.pone.0010604

**Published:** 2010-05-13

**Authors:** Marko Roblek, Stefan Schüchner, Veronika Huber, Katrin Ollram, Sylvia Vlcek-Vesely, Roland Foisner, Manfed Wehnert, Egon Ogris

**Affiliations:** 1 Department of Medical Biochemistry, Max F. Perutz Laboratories, Medical University of Vienna, Vienna, Austria; 2 Institute of Human Genetics, Ernst-Moritz-Arndt-University, Greifswald, Germany; Tulane University Health Sciences Center, United States of America

## Abstract

**Background:**

Disease-linked missense mutations can alter a protein's function with fatal consequences for the affected individual. How a single amino acid substitution in a protein affects its properties, is difficult to study in the context of the cellular proteome, because mutant proteins can often not be traced in cells due to the lack of mutation-specific detection tools. Antibodies, however, with their exquisite epitope specificity permit the detection of single amino acid substitutions but are not available for the vast majority of disease-causing mutant proteins. One of the most frequently missense-mutated human genes is the *LMNA* gene coding for A-type lamins. Mutations in *LMNA* cause phenotypically heterogenous, mostly autosomal-dominant inherited diseases, termed laminopathies. The molecular mechanisms underlying the phenotypic heterogeneity of laminopathies, however, are not well understood. Hence, the goal of this study was the development of monoclonal antibodies specific for disease-linked point-mutant A-type lamins.

**Methodology/Principal Findings:**

Using two different approaches of antigen presentation, namely KLH-coupled peptides and the display of a complete protein domain fused to the Hepatitis B virus capsid protein, we developed monoclonal antibodies against two disease-associated lamin A/C mutants. Both antibodies display exquisite specificity for the respective mutant proteins. We show that with the help of these novel antibodies it is now possible for the first time to study specifically the properties of the mutant proteins in primary patient cells in the background of wild-type protein.

**Conclusions:**

We report here the development of two point-mutant specific antibodies against A-type lamins. While synthetic peptides may be the prime choice of antigen, our results show that a given target sequence may have to be presented in alternative ways to ensure the induction of a mutant-specific immune response. Point-mutant specific antibodies will represent valuable tools for basic and clinical research on a number of hereditary as well as acquired diseases caused by dominant missense mutations.

## Introduction

Currently, the NCBI dbSNP database has annotated more than 25 million human single nucleotide polymorphisms (SNPs) (http://www.ncbi.nlm.nih.gov/snp/), and it is these SNPs, which are responsible for the phenotypic differences between human individuals. Non-synonymous (ns) SNPs, which result in an amino acid change in the encoded protein, or SNPs in gene regulatory regions can be associated with genetic diseases or an altered susceptibility to disease. A number of bioinformatic studies have used evolutionary and structural approaches to predict the effect of nsSNPs (or missense mutations) on protein structure and function (http://coot.embl.de/PolyPhen/ or http://mmb2.pcb.ub.es:8080/PMut/) [1], [2], [3], [4], [5]. However, a direct experimental analysis of the mutant protein in patient cells has proven very difficult in cases, where the wild type and the mutant protein are present in the same cell, such as in autosomal dominant inherited diseases, largely because of the lack of tools, which specifically recognize the mutant protein within the proteome context and which would allow to study the effect of the SNP/mutation on the protein function within this network. Hence, for many diseases our knowledge of how mutations change protein structure and function - e.g. its enzymatic activity, sub-cellular localization, or interactions with other proteins – and why and how this is linked to a certain disease phenotype, is still limited to studies based on the ectopic expression of a tagged, mutant protein in non-diseased cells. Thus, we need better and highly specific research tools, which allow to distinguish between the wild-type and mutant protein. Monoclonal antibodies with their unrivalled specificity for defined epitopes can fulfill these demands. Moreover, besides their use in basic research for better understanding the molecular mechanisms of the disease pathogenesis, monoclonal antibodies can also be applied for the diagnosis and prognosis of diseases as well as for therapeutic interventions. Hence, point-mutant specific monoclonal antibodies may not only represent extremely powerful research tools to study somatic and inherited genetic diseases, but could also emerge as valuable medical tools in the future.

**Figure 1:**
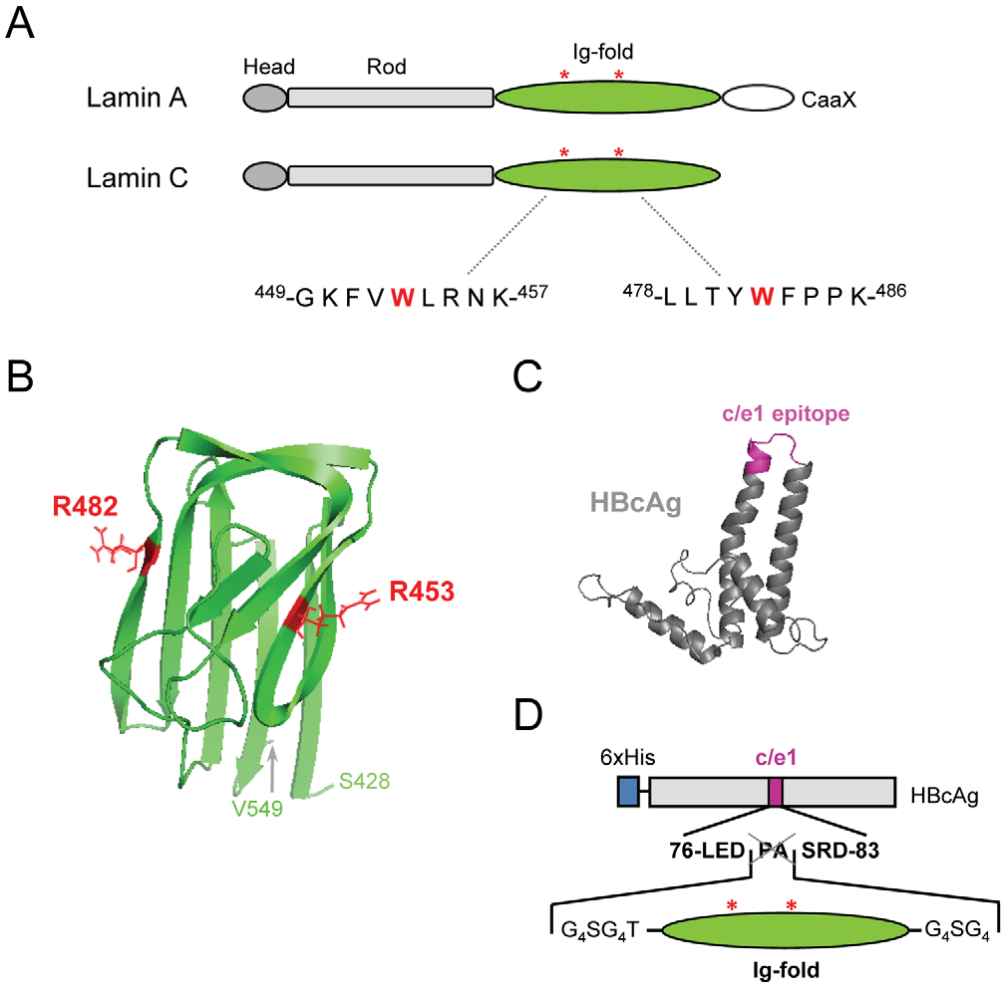
Schematic presentation of lamin A/C and the HBcAg-Ig-fold fusion protein. (**A**) Tripartite structure of Lamin A and Lamin C consisting of an N-terminal head domain, a long central alpha-helical rod domain, and a globular C-terminal Ig-fold domain. Lamin A contains a CaaX signal motif for post-translational processing at its C-terminus. The two asterisks highlight the two mutations within the Ig-fold domain, R453W and R482W, respectively, which were chosen as antigens. The amino acid sequences comprising the respective mutant amino acids are highlighted. (**B**) Structure of the lamin A/C Ig-fold. The NMR structure obtained from Krimm et al. [11] was modified with the PyMOL Molecular Viewer program. The position and the side chains of the amino acids Arg453 and Arg482 are highlighted in red, and the first and the last amino acid of the Ig-fold (Ser428 and Val549, respectively) are indicated. (**C**) Structure of HBcAg. The crystal structure obtained from Wynne et al. [50] was modified with the PyMOL Molecular Viewer program. The immunodominant B-cell epitope c/e1 is highlighted in purple. (**D**) Recombinant HBcAg-Ig-fold protein. The Ig-fold, either in the wild-type sequence or with the R453W or R482W point mutation (indicated by an asterisk) was cloned into the c/e1 epitope of the His-HBcAg protein (aa 1–149), substituting amino acids Pro79 and Ala80. The Ig-fold is flanked by flexible glycine-rich linkers to allow proper folding of the inserted Ig-fold as well as of the HBcAg protein.

Laminopathies represent a group of rare human hereditary diseases, which are caused by mutations in genes encoding components of the nuclear lamina, including the A- and B-type lamins as well as lamin-associated proteins, e.g. emerin, lamina associated polypeptide (LAP) 2α, or lamin B receptor (LBR) [6], [7]. Today, at least 13 distinct laminopathies are known, which display heterogenous phenotypes and include skeletal and/or cardiac muscle dystrophies (e.g. Emery-Dreifuss muscular dystrophy - EDMD), lipodystrophies (e.g. Dunnigan-type familial partial lipodystrophy - FPLD), peripheral neuropathies, and accelerated ageing syndromes [6], [8], [9]. In the *LMNA* gene alone, which codes for the A-type lamins A and C, more than 200 mutations, predominantly inherited in an autosomal dominant fashion and in most cases single point missense mutations, have been described. Interestingly, these mutations are found in all exons with no apparent hot-spot clusters (Leiden Open Variation Database (http://www.dmd.nl/lmna_seqvar.html)). Lamin A and lamin C share the first 566 amino acids and display the characteristic intermediate filament protein tripartite structure with a short N-terminal head domain, a central α-helical rod domain and a C-terminal globular domain, which in case of lamin A/C includes an Ig-like fold (Figure 1A) [10], [11], [12]. While there are no mutation hot-spot regions apparent in the *LMNA* gene, missense mutations affecting two residues, which are both located within the Ig-fold domain, have been found in a relatively large percentage of laminopathy patients: first, an exchange of arginine 453 by tryptophan (R453W), which was detected in 16% of autosomal-dominant (AD-) EDMD cases (corresponding to 11% of all laminopathy cases), and second, the substitution of arginine 482 by either tryptophan, glutamine, or leucine (R482W/Q/L), which is responsible for more than 80% of FPLD cases (and 13% of all laminopathy cases) [13].

During the last decade few genes have received as much attention from the scientific community as the *LMNA* gene. Originally thought to play solely a structural role in order to maintain nuclear shape, A-type lamins have now emerged as crucial players of several functions in the nucleus, ranging from transcriptional control and the organization of heterochromatin to the regulation of various cell signaling pathways [14]. Nevertheless, it remains largely elusive, how mutations in a ubiquitously expressed gene can cause tissue restricted pathogenic phenotypes. Several possible explanations have been suggested, including changes in lamin A/C dependent signaling pathways [15], [16], increased sensitivity to mechanical stress [17], impaired differentiation [18], or alterations in lamina-mediated gene expression [19]; however, different mechanisms (or a combination thereof) might be responsible depending on the type of affected tissue. Alternatively, impaired tissue regeneration due to deregulated adult stem cells may be responsible for some of the pathological conditions seen in laminopathy patients [20]. Since the vast majority of *LMNA* mutations are inherited in a dominant fashion, resulting in the side-by-side expression of wild-type and mutant lamin A/C, researchers so far had to rely on studies using either knock-out, knock-in or transgenic animals, or using non-diseased cells, in which a certain tagged lamin A/C mutant was ectopically expressed. Interestingly, most studies were based on the ectopic expression of mutant lamin A alone without concomitant expression of the respective lamin C mutant. With the exception of mutations in the lamin A-unique far C-terminus, such an approach creates a cellular environment, which does not mirror the “real” situation in patient cells, where both A-type lamins are affected by a mutation in the *LMNA* gene. Furthermore, while several *Lmna* mouse models mimic the phenotypes of laminopathies, such phenotypes manifest themselves only under homozygous conditions [21]. Thus, antibodies specifically recognizing mutant lamin A/C proteins would be highly valuable research tools to study the molecular mechanisms of laminopathies in primary human patient cells and tissues.

In this work, we generated two mouse monoclonal antibodies, which specifically detect disease associated lamin A/C mutants but do not cross-react with the wild-type counterpart. We show that the design of the antigen, by which a certain mutated amino acid is displayed, can be crucial for the induction of a specific immune response. While an R453W specific antibody was generated against a synthetic peptide, a peptide antigen failed to elicit an R482W mutant specific humoral immune response. Rather, the presentation of tryptophan 482 in the context of the whole C-terminal Ig-fold domain of lamin A/C led to the development of an R482W specific antibody. The novel antibodies presented here, whose specificity was evaluated by Western blotting, competition experiments, immunoprecipitation, and immunofluorescence microscopy assays, can now be applied to selectively study features of the mutant A-type lamins in primary patient cells.

## Materials and Methods

### Ethics Statement

The maintenance of mice and experimental procedures have been conducted according to the Austrian Animal Experiments Act and have been approved by the Austrian Federal Ministry of Science and Research (GZ 66.009/101-BrGT/2005 and GZ 66.009/61-C/GT/2007) and the animal experiments ethics committee of the Medical University of Vienna. For the primary human skin fibroblasts written informed consent was provided by the patients for the investigation, sample and data management under a protocol (BB53/07) approved August 03, 2007 by the University of Greifswald ethics board according to the principles expressed in the Declaration of Helsinki.

### Cloning of expression constructs

The HBcAg cDNA for the HBcAg-Lamin A/C Ig-fold expression plasmids was derived from the HBcAg-GFP expression vector, pET28a-c149GFP (a gift from Michael Nassal, Freiburg). pET28a-c149GFP was cut with NcoI/KpnI resulting in a fragment coding for the N-terminus (aa1–78) of HBcAg followed by a flexible glycine-rich linker (amino acid sequence: GGGGSGGGGT). This N-terminal fragment of HBcAg was inserted together with a new multiple cloning site (annealed oligos MCS_fw and MCS_rev; containing the following restriction sites: SnaBI, SphI, BsrGI, HindIII) into the bacterial expression vector pB'His-NP cut with NcoI/SphI to give pB'His-HBcAg-Nterm. The DNA sequence coding for a second flexible glycine-rich linker (amino acid sequence: GGGGSGGGG) followed by the C-terminus of HBcAg (aa81–149) was cut from pET28a-c149GFP with BsrGI/HindIII and cloned into pB'His-HBcAg-Nterm to give pB'His-HBcAg-linker. The coding sequence of the wild-type, the R453W, and the R482W mutant lamin A/C Ig-folds was amplified by PCR from the respective pSVK3-LaminA plasmids [22] with primers Igfold_fw and Igfold_rev. The PCR products were cut with KpnI/BsrGI and cloned into pB'His-HBcAg-linker to give pB'His-HBcAg-Ig-fold wt, pB'His-HBcAg-Ig-fold R453W, and pB'His-HBcAg-Ig-fold R482W.

pB'His-HBcAg-Lamin A 478–487 R482W was cloned by inserting the annealed oligos 478–487 R482W_fw and 478–487 R482W_rev into pB'His-HBcAg-linker. pB'His-HBcAg-Lamin A 472–492 R482W was cloned by inserting the annealed oligos 472–492 R482W_fw and 472–492 R482W_rev into pB'His-HBcAg-linker (both were inserted via KpnI/BsrGI). The coding sequence for aa 458–503, or 438–523 was amplified by PCR from pB'His-HBcAg-Ig-fold R482W with the primers 458_fw and 503_rev, or 438_fw and 523_rev, respectively, and inserted into pB'His-HBcAg-linker via KpnI/BsrGI. The oligonucleotide sequences are depicted in Table 1. The sequence of all expression constructs was confirmed by sequencing.

**Table 1:** Oligonucleotides.

MCS_fw	5′-CTACGTAGCATGCATGTACAACGAAGCTTACATG-3′
MCS_rev	5′-TAAGCTTCGTTGTACATGCATGCTACGTAGGTAC-3′
Ig-fold_fw	5′-CACATTGGTACCTTCTCACAGCACGCACGC-3′
Ig-fold_rev	5′-GAAATTGTACATGCGCACCAGCTTGCGCATGG-3′
478–487 R482W_fw	5′-CTTGCTGACTTACTGGTTCCCACCAAAGTTCAT-3′
478–487 R482W_rev	5′-GTACATGAACTTTGGTGGGAACCAGTAAGTCAGCAAGGTAC-3′
472–492 R482W_fw	5′-CCAGAATGGAGATGATCCCTTGCTGACTTACTGGTTCCCACCAAAGTTCACCCTGAAGGCTGGGAT-3′
472–492 R482W_rev	5′-GTACATCCCAGCCTTCAGGGTGAACTTTGGTGGGAACCAGTAAGTCAGCAAGGGATCATCTCCATTCTGGGTAC-3′
458_fw	5′-CACATTGGTACCTCCAATGAGGACCAG-3′
503_rev	5′-GAAATTGTACATCCCAGCTCCTGCAGC-3′
438_fw	5′-CACATTGGTACCGGGCGCGTGGCCGTG-3′
523_rev	5′-GAAATTGTACATCCCGCAGCCCCAGGT-3′

### Expression and purification of recombinant His-HBcAg Ig-fold fusion proteins

6xHis-tagged recombinant proteins were expressed in the bacterial strain Rosetta(DE3)pLysS (Novagen). Expression was induced with 1mM IPTG for 3.5 hours at 37°C or room temperature (RT). The bacterial cells were lysed by sonication (3×30 sec with 20 sec breaks between the sonication steps) on ice in 25 mM Tris pH 8.4 containing PMSF and aprotinin. The 6xHis-tagged proteins were purified from the soluble fraction of the lysate by incubation with Ni-NTA Agarose beads (Qiagen) o/n at 4°C. The beads were washed 4x with 25 mM Tris pH 8.4 (plus PMSF and aprotinin) and 5x with washing buffer (50 mM NaH_2_PO_4_; 300 mM NaCl; 20 mM imidazole, pH 8.0, PMSF, aprotinin). Proteins were eluted with elution buffer (50 mM NaH_2_PO_4_; 300 mM NaCl; 250 mM imidazole, pH 8.0, PMSF, aprotinin) for 1 hour at 4°C or RT.

#### Peptides

The following keyhole limpet hemocyanin (KLH)-coupled peptides (purchased from piCHEM, Graz, Austria) were used for immunizations: R453W: Ac-^449^GKFVWLRNK^457^-C-NH_2_; R482W: Ac-C-^478^LLTYWFPPK^486^-NH_2_).

### Immunization of mice and fusion of splenocytes with myeloma cells

50 µg of antigen per mouse and immunization were mixed with adjuvant at a ratio of 1∶1 and injected subcutaneously. After three to four immunizations, a final boost of 50 µg of antigen without adjuvant was injected into the tail vein. Four days after the final immunization the splenocytes were fused with the myeloma cell line X63Ag8.653 according to standard protocols using polyethylene glycol [23]. Hybridoma supernatants were screened for the presence of antibodies by Western blotting after 7 days. Positive single clones were obtained by limited dilution of mixed hybridoma clones.

### Cell lines

To generate cell lines stably expressing Flag-tagged lamin A wt/R453W/R482W, HeLa cells (ATCC No. CCL-2; www.atcc.org) were co-transfected with pSVK3-Flag-LaminA wt/R453W/R482W and pTK-Hyg (Clontech; conferring hygromycin B resistance) at a ratio of about 15∶1 using lipofectamine 2000 (Invitrogen) according to the manufacturer's guidelines. Stably expressing cells were selected in the presence of 175 µg/ml of Hygromycin B. X63Ag8.653 myeloma cells (ATCC No. CRL-1580; www.atcc.org), HeLa cells and HeLa cells ectopically expressing the respective Flag-tagged versions of Lamin A (hereafter termed HeLa Flag-Lamin A) - HeLa Flag-Lamin A wt, HeLa Flag-Lamin A R453W, and HeLa Flag-Lamin A R482W - were cultured at 37°C in an atmosphere of 7.5% CO_2_ (HeLa) or 5% CO_2_ (X63Ag8.653) in DMEM (Sigma) supplemented with 10% fetal bovine serum (FBS, Gibco), 1% Penicillin/Streptomycin (Sigma), 2mM Glutamax (Gibco) and 1mM Na-Pyruvate (Sigma). HeLa Flag-Lamin A cells were cultured in the presence of 175 µg/ml Hygromycin B (Calbiochem). Hybridoma clones were selected in hypoxanthine-aminopterin-thymidine (HAT) medium consisting of DMEM, 10% FBS, 5% BM-Condimed H1 Hybridoma Cloning Supplement (Roche), 100 U/ml Penicillin, 0.1 mg/ml Streptomycin, 2 mM Glutamax, 1 mM Na-Pyruvate, and HAT supplement (Gibco).

#### Primary human fibroblasts

Wild-type, R453W (G-8626), and R482W (G-9956) primary human skin fibroblasts [24] were provided by Manfred Wehnert and were cultured at 37°C in an atmosphere of 7.5% CO_2_ in DMEM supplemented with 10% FBS, 100 U/ml Penicillin, 0.1 mg/ml Streptomycin, 2 mM Glutamax and 1 mM Na-Pyruvate. All experiments were performed with wild-type fibroblasts during passages 32–38, R453W fibroblasts during passages 30–34, and R482W fibroblasts during passages 28–32.

#### Western blotting/Dot blotting

Cells were lysed in pre-heated Laemmli buffer to ensure complete solubilization of nuclear lamina proteins. Proteins were separated by SDS-PAGE, followed by blotting onto a nitrocellulose membrane (Whatman, 0.2 µm). For dot blots, 1 µg of peptides or recombinant proteins were spotted onto a PVDF membrane (Millipore), and equal spotted amounts were confirmed by Ponceau S staining. The membranes were blocked with 3% non fat dry milk (NFDM) in PBS-Tween 20 (0.05%) for 1 hour at RT and incubated with primary antibody in 0.5% NFDM/PBS-Tween 20 o/n at 4°C. Incubation with secondary peroxidase conjugated anti-mouse antibody (AffiniPure Goat Anti Mouse IgG (Fcγ fragment) specific antibody, Jackson ImmunoResearch, 1∶5000) was performed for 1 hour at RT, followed by incubation with Western Lightning ECL solution (PerkinElmer) as suggested by the manufacturer. The signals were detected by a Super HR-HA 30 film (Fuji). Lamin A and C were detected with mouse monoclonal antibody, clone 4C11, which was raised against the wild-type Ig-fold domain. Mature lamin A was detected with a mouse monoclonal antibody, clone 4A4, which was raised against a C-terminal peptide of mature lamin A. Commercially available antibodies were: anti Prelamin A (1∶500, C-20, Santa Cruz), anti Lamin A/C (1∶1000, N-18, Santa Cruz), anti Flag (1∶10000, M2, Sigma,) and anti β-actin (1∶5000, clone AC-74, Sigma).

### Immunofluorescence

Cells were seeded onto glass cover-slips, and cultured o/n in the appropriate medium. The cover-slips were washed 2x with PBS and fixed in 3,7% formaldehyde/PBS for 15 min, quenched with 50 mM NH_4_Cl/PBS for 10 min, permeabilized with 0.2% Triton X-100/PBS for 10 min, blocked with 0.2% gelatine/PBS for 1 hour and then incubated with the primary antibody diluted in 0.2% gelatine/PBS for 2 hours. For staining with the anti R453W antibody, permeabilization was carried out in the presence of 0.1% SDS. Commercially available primary antibodies included: goat anti-LA/C (1∶200; N-18, Santa Cruz) and anti Flag (1∶1000; M2, Sigma). Incubation with secondary antibodies, Alexa Fluor 594 goat anti-mouse IgG (H+L) (1∶500, Molecular Probes), Alexa Fluor 488 donkey anti-mouse (H+L) (1∶500, Molecular Probes), or donkey anti-goat IgG (H+L) Texas Red (1∶200, Jackson ImmunoResearch) was done for 1 hour. The DNA was counterstained with Hoechst 33342 and the cover-slips were mounted with Vectashield (Vector Laboratories). All steps were performed at RT. Pictures were taken with a Zeiss LSM 510 Meta confocal microscope using the corresponding LSM 510 META software. Relative fluorescence intensity profiles were determined using the same software. For presentation, images were adjusted for brightness and contrast.

### Peptide/Ig-fold competition assay

For competition with peptide, the R453W antibody was diluted in 0.5% NFDM/PBS-Tween 20 as appropriate for Western blotting (1∶100) and a fixed amount of antibody was incubated with increasing amounts of the peptide (0,44pmol, 2,2pmol, 11pmol, 55pmol) o/n at 4°C. Nitrocellulose membranes were then incubated for 2 hours at RT with the respective antibody-peptide solutions, and subjected to Western blot detection as described above. For competition with recombinant protein, fixed amounts of His-HBcAg-Ig-fold fusion proteins (wt or R482W; 0,13nmol) were incubated with decreasing amounts of R482W antibody (38 µl, 3,8 µl, 0.38 µl of hybridoma supernatant) in 0.5% NFDM/PBS-Tween 20 for 2 hours at RT. After the competition, all antibody-HBcAg-Ig-fold solutions were then further diluted appropriately in 0.5% NFDM/PBS-Tween 20 to give the same antibody dilution (1∶250) for Western blotting and used for Western blot detection as described above.

### Native cell lysates and immunoprecipitation

Cells were lysed in ice-cold cell lysis buffer (20 mM Tris pH 7.5, 150 mM NaCl, 1 mM EDTA, 1mM EGTA, 1% Triton X-100, PMSF, Aprotinin, complete protease inhibitor cocktail (Roche)) for 5 min on ice, followed by sonication on ice (3×5 seconds). Antibodies (anti R453W, anti R482W, anti lamin A/C 4C11) were covalently cross-linked to protein A sepharose beads (GE Healthcare). Immunoprecipitation was performed for 1 hour at 4°C (anti R482W and anti lamin A/C 4C11), or for 1 hour at RT in the presence of 0.25% SDS (anti R453W). After washing, the beads were boiled in Laemmli buffer and immunoprecipitated proteins were separated by SDS-PAGE for subsequent detection by Western blotting.

### Sub-cellular fractionation

Fractionation was performed according to published protocols [25], [26]. Briefly, cells were resuspended in 1 ml Buffer F1 (20 mM Tris pH 7.6, 50 mM β-Mercaptoethanol, 0.1 mM EDTA, 2 mM MgCl_2_, Aprotinin, PMSF, complete protease inhibitor cocktail (EDTA free)) per 5×10^6^ cells and incubated for 2 min at RT, followed by incubation on ice for 10 min. NP-40 was added to a final concentration of 1% (v/v) and the solution was homogenized by repeated pipetting through a yellow Gilson tip. The nuclei were pelleted by centrifugation at 600 g for 5 min at 4°C, separated from the cytoplasmic fraction and washed 2x in Buffer F1 + 1% NP-40 (v/v). Nuclei were resuspended in 500 µl hypotonic buffer (10 mM HEPES pH 7.4, 10 mM NaCl, 5 mM MgCl_2_, 1 mM EGTA, 1 mM DTT, 8.5% sucrose (w/v), PMSF, Aprotinin, complete protease inhibitor cocktail (EDTA free)) per 10^7^ nuclei. For low salt extraction, NaCl was added to a final concentration of 50 mM. For high salt/detergent extraction, NaCl was added to a final concentration of 200 mM and Triton X-100 to a final concentration of 1% (v/v). The samples were incubated for 20 min on ice and subsequently centrifuged at 16000 g for 30 min at 4°C. Soluble and insoluble fractions were boiled in Laemmli buffer, and the proteins were separated by SDS-PAGE and detected by Western blot analysis.

Materials will be sent out upon request to EO or SS.

## Results

We sought to develop antibodies against two disease-linked lamin A/C mutants, R453W and R482W, because of their relatively frequent incidence and the gross change in the amino acid side chain. To ensure a high chance of stimulating a mutant-specific immune response, two complementary approaches of antigen presentation were pursued: first, we used short synthetic peptides of nine amino acids coupled to KLH with the respective tryptophan residue in the center position (Figure 1A). Second, the lamin A/C Ig-fold domain (aa 430–545) - either with the wild-type sequence or containing the R453W or the R482W substitution - was inserted into the hepatitis B virus capsid protein (HBcAg) that lacks the C-terminal DNA/RNA-binding region (Figure 1D).

HBcAg is a very strong immunogen containing potent T helper cell epitopes and the immunodominant c/e1 B cell epitope surrounding amino acid 80 (Figure 1C) [27]. In addition, HBcAg can also act as a T-cell independent antigen due to its ability to form multimers, which allows the simultaneous cross-linking of sufficient surface B-cell receptors to activate naive B-lymphocytes without the help of T-lymphocytes [28]. It has been shown that a strong and specific humoral immune response can be mounted against heterologous proteins like the green fluorescence protein (GFP) or the outer surface protein A (OspA) from *Borrelia burgdorferi* when inserted into HBcAg at the c/e1 epitope [28], [29]. We reasoned that the compact structure of the lamin A/C Ig-fold together with the fact, that the N- and C-termini of this domain are in close spatial proximity (Figure 1B) [10], [11], should allow the expression of a recombinant fusion protein in bacteria, in which the lamin A/C Ig-fold would adapt its native conformation and be situated at the position of the highly immunogenic c/e1 B cell epitope of the hepatitis antigen (Figure 1D). Interestingly, arginine 482 lies within a short stretch of amino acids, which have been shown to represent a major surface epitope of lamin A [30]. We therefore hypothesized that, although the Ig-fold comprises 116 amino acids and most likely contains a number of potential B cell epitopes [31], [32], at least the FPLD-associated R482W mutant could represent a likely target when presented in the context of the whole native Ig-fold domain.

### Lamin A/C R453W antibody is specific for the EDMD-causing point-mutant R453W

**Figure 2:**
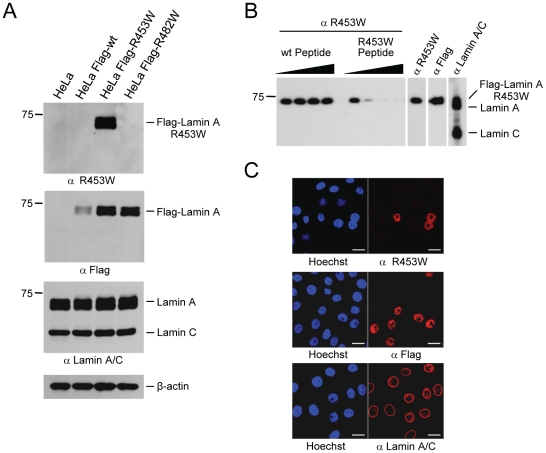
R453W antibody is specific for the EDMD associated lamin A/C R453W point-mutant. (**A**) HeLa, HeLa Flag-Lamin A wt, HeLa Flag-Lamin A R453W, and HeLa Flag-Lamin A R482W cell lysates were separated by 7.5% SDS-PAGE and analyzed by Western blotting with the mouse monoclonal antibodies anti R453W (panel I), anti Flag M2 (panel II), anti Lamin A/C 4C11 (panel III), and β-actin (panel IV). (**B**) Peptide competition of R453W antibody. Binding of R453W antibody to Flag-Lamin A R453W was competed with increasing amounts (0.44, 2.2, 11, and 55pmol) of mutant “R453W Peptide” (NH_2_-^449^GKFVWLRNK^457^-COOH) or wild-type “wt Peptide” (NH_2_-^449^GKFVRLRNK^457^-COOH).Competition efficiency was examined by 7.5% SDS-PAGE/Western blot analysis of HeLa Flag-Lamin A R453W cell lysates. Control antibodies were: anti R453W not subjected to competition, anti Flag M2, anti Lamin A/C 4C11. (**C**) Immunofluorescence of a mixed population of HeLa and HeLa Flag-Lamin A R453W cells, seeded at a ratio of 3∶1. Cell staining was performed with anti R453W (top panel), anti Flag M2 (middle panel), and anti Lamin A/C 4C11 (bottom panel). DNA was counterstained with Hoechst 33342. Bar scale, 20 µm.

A monoclonal antibody was generated from a Balb/c mouse immunized with the R453W-KLH peptide (aa 449–457) and its specificity for the R453W point-mutant was examined by Western blotting using lysates of HeLa cells as well as HeLa cells expressing Flag-lamin A wt, Flag-lamin A R453W, or Flag-lamin A R482W, respectively (Figure 2A, top panel). The R453W antibody recognized specifically the Flag-lamin A R453W mutant, but did not cross-react with Flag-lamin A wt or -R482W. The fact that Flag-lamin A wt was not detected by the R453W antibody could be due to the significantly lower expression levels of Flag-lamin A wt in these cells compared to the two mutants as revealed by staining with a Flag-specific antibody (Figure 2A, second panel). However, the R453W antibody did not detect any endogenous wt lamin A or lamin C, which were detected using a monoclonal lamin A/C antibody, clone 4C11 (Figure 2A, third panel). This antibody had been raised against the wt lamin A/C Ig-fold and recognizes the wt and mutant lamin A/C proteins, albeit the R453W mutant to a lesser extent (Figure S1). Taken together, these findings strongly suggested that the R453W antibody was specifically recognizing the respective mutant form of lamin A.

The specificity of the antibody was further examined by a peptide competition assay (Figure 2B). The peptide containing the R453W substitution and the corresponding wild-type peptide were tested for their ability to interfere with the binding of the R453W antibody to Flag-lamin A R453W by pre-incubating the antibody with increasing amounts of peptides followed by Western blotting. Detection of the Flag-tagged lamin A R453W mutant was strongly reduced by pre-incubation of the antibody with 2.2pmol of the mutant R453W peptide, and completely abolished by pre-incubation with 11pmol of the mutant peptide. In contrast, 55pmol (and even amounts of up to 1nmol, data not shown) of the wild-type peptide could not interfere with detection of Flag-lamin A R453W, indicating that the point-mutant specific antibody did not have any detectable affinity for the wild-type peptide.

We next examined whether the R453W antibody would also specifically detect the mutant protein in immunofluorescence analysis (IF; Figure 2C). HeLa and HeLa Flag-lamin A-R453W cells were seeded at a ratio of 3∶1, and subsequently stained with the indicated antibodies. The general lamin A/C antibody, 4C11, stained all cells (lower panel). In contrast, a Flag-tag antibody stained only a sub-population of cells (middle panel). Similarly, the R453W antibody stained just approximately one quarter of all cells (upper panel), suggesting that also by IF it was only detecting the ectopic Flag-lamin A mutant.

### Lamin A/C R482W antibody is specific for the FPLD-causing point-mutant R482W

Substitutions of arginine 482 are responsible for 80% of FPLD cases and 13% of all laminopathies. Thus, we sought to develop a second point-mutant specific antibody against the lamin A/C R482W mutant by immunizing mice with the respective KLH-coupled peptide encompassing amino acids 478–486. Interestingly, despite of the short size of the peptide antigen several animals developed “general” antibodies against lamin A/C, but none showed a mutant-specific immune response by Western blotting (data not shown).

Therefore we applied an alternative way of antigen presentation, using a native, recombinant HBcAg Ig-fold R482W fusion protein. By this approach, one would expect that the immunized animals develop antibodies against numerous B-cell epitopes on the surface of the Ig-fold [31], [32]. Indeed, the polyclonal sera of most of the immunized mice recognized endogenous lamin A/C as well as ectopic Flag-lamin A R482W in HeLa cell lysates (data not shown). While we were able to confirm the presence of R482W mutant-specific antibodies within the polyclonal mixture by means of fusing splenocytes from such animals with myeloma cells and seeding the cell-hybrids at low density, none of the mutant-specific antibody producing hybridoma clones was stable (data not shown). However, one out of 8 mice immunized with the HBcAg Ig-fold R482W fusion protein developed a polyclonal immune response, which was predominantly detecting the lamin A/C R482W mutant (data not shown), and a monoclonal antibody was generated from this mouse.

**Figure 3:**
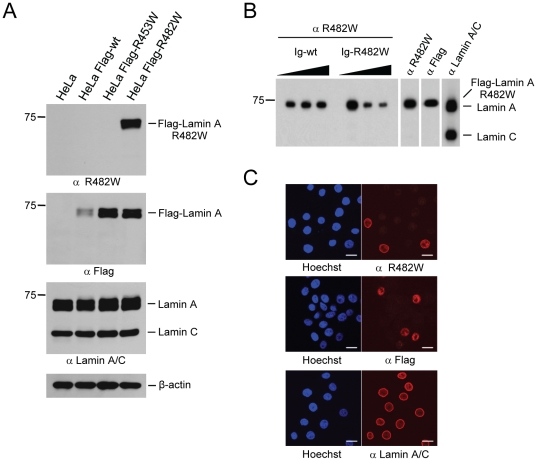
R482W antibody is specific for the FPLD-associated lamin A/C R482W point-mutant. (**A**) 7.5% SDS-PAGE of HeLa, HeLa Flag-Lamin A wt, HeLa Flag-Lamin A R453W, and HeLa Flag-Lamin A R482W cell lysates followed by Western blot analysis with the mouse monoclonal antibodies anti R482W (panel I), anti Flag M2 (panel II), anti Lamin A/C 4C11 (panel III), and β-actin (panel IV). (**B**) Ig-fold competition of R482W antibody. Binding of anti R482W to Flag-Lamin A R482W was competed with recombinant HBcAg protein containing either the wild-type Ig-fold, “Ig-wt”, or the mutant Ig-fold, “Ig-R482W”, at increasing molar ratios. Competition efficiency was examined by 7.5% SDS-PAGE/Western blot analysis of HeLa Flag-Lamin A R482W cell lysates. Control antibodies were: anti R482W not subjected to competition, anti Flag M2, anti Lamin A/C 4C11. (**C**) Immunofluorescence of a mixed population of HeLa and HeLa Flag-Lamin A R482W cells, seeded at a ratio of 3∶1. Cells were stained with anti R482W (top panel), anti Flag M2 (middle panel), and anti Lamin A/C 4C11 (bottom panel). DNA was counterstained with Hoechst 33342. Bar scale, 20 µm.

We first tested the specificity of the R482W monoclonal antibody by Western blotting using again lysates from HeLa, HeLa Flag-lamin A wt, HeLa Flag-lamin A R453W, and HeLa Flag-lamin A R482W cells. The R482W antibody specifically detected Flag-lamin A R482W with no cross-reactivity to the other two Flag-tagged lamin A proteins (Figure 3A, top panel; the Flag, lamin A/C and β-actin blots in Figure 3A are identical to Figure 2A). In addition, also endogenous lamin A and C were not detected. We have to note, however, that when used under sub-optimal conditions, e.g. large amounts of protein lysate paired with high concentrations of antibody, the R482W antibody cross-reacted very weakly with endogenous wild type lamin A/C, indicating a very low but detectable affinity of the R482W antibody for the wild-type proteins (data not shown).

Similar to the R453W antibody, we wanted to affirm the specificity of the R482W antibody in a competition assay using wild-type and mutant peptides corresponding to amino acids 478 to 486 of lamin A/C. However, we did not observe any competition (data not shown) indicating that the R482W antibody did not bind to such short peptides. Thus, a competition assay was performed, in which the mutant R482W Ig-fold and the corresponding wild-type Ig-fold (both purified under native conditions as HBcAg fusion proteins) were tested for their ability to sequester the antibody and interfere with detection of Flag-lamin A R482W by Western blot analysis (Figure 3B). Pre-incubation at increasing ratios of mutant R482W Ig-fold to antibody progressively reduced detection of Flag-lamin A R482W. On the other hand, while the Western blot signal was generally slightly weaker after incubation with the wild-type Ig-fold compared to the mock treated antibody control, increasing ratios of wild-type Ig-fold to antibody did not abolish detection of Flag-lamin A R482W, suggesting that the HBcAg-Ig-fold wt could not sequester the R482W-specific antibody. These findings further highlighted the high specificity of the lamin A/C R482W antibody for the mutant protein.

Finally, the usefulness of the antibody for immunofluorescence studies was again tested using HeLa and HeLa Flag-lamin A R482W cells that were seeded at a ratio of 3∶1 and stained with the indicated antibodies (Figure 3C). While the general anti-lamin A/C antibody stained all cells, only sub-populations of cells were stained by the Flag-tag antibody or the R482W antibody, respectively, again strongly suggesting that the R482W antibody was specifically recognizing only the ectopic mutant protein.

### The R482W antibody recognizes a conformation-dependent discontinuous epitope

**Figure 4:**
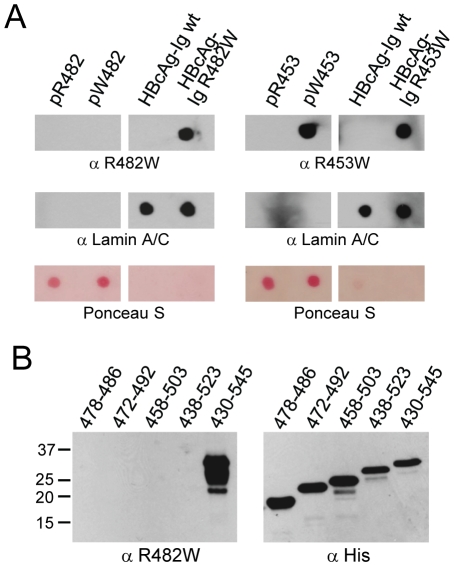
R482W antibody recognizes a discontinuous epitope in the lamin A/C Ig-fold. (**A**) Equal amounts of peptide pR482 (^472^QNGDDPLLTYRFPPKFTLKAG^492^), pW482 (^472^QNGDDPLLTYWFPPKFTLKAG^492^), pR453 (^449^GKFVRLRNK^457^), pW453 (^449^GKFVWLRNK^457^) and equal amounts of recombinant HBcAg Ig-fold wt, HBcAg Ig-fold R453W, and HBcAg Ig-fold R482W were spotted onto PVDF membranes. Blots were incubated either with anti R482W antibody (upper left panels) or with anti R453W antibody (upper right panels). Anti lamin A/C (middle panels) and Ponceau S staining (bottom panels) confirmed equal spotting of recombinant HBcAg Ig-fold proteins or peptides, respectively. (**B**) 12.5% SDS-PAGE of bacterial lysates expressing His-tagged HBcAg Ig-fold R482W fragments of increasing size followed by Western blot analysis with R482W antibody (left panel). Equal amounts of recombinant proteins were confirmed by incubation with anti-His antibody (right panel). Numbers correspond to amino acids of lamin A/C.

The results of the competition experiments with the R482W antibody indicated that the epitope of this antibody may comprise either a linear continuous sequence that is longer than the 9-mer peptide used for the competition experiment, or a discontinuous, conformational epitope in the natively folded lamin A/C Ig-fold. The R453W antibody, on the other hand, recognized a continuous epitope as suggested by the efficient blocking of antigen recognition by a linear 9-mer peptide containing the R453W mutation. To further examine the binding abilities of the two antibodies, we carried out a dot-blot assay with wild-type and mutant peptides or entire Ig-fold domains (Figure 4A). Consistent with the results of the competition experiments, the R482W antibody specifically detected the R482W mutant only if displayed in the Ig-fold, whereas the R453W antibody recognized the mutant displayed in the peptide as well as in the Ig-fold. In order to map the R482W epitope we examined the ability of the antibody to detect by Western blot Ig-fold R482W fragments of increasing size inserted into the HBcAg protein (Figure 4B). Strikingly, the R482W antibody detected only the full-length mutant Ig-fold suggesting that the presence of the whole Ig-fold domain was required for recognition by the R482W antibody. This finding strongly indicated that the R482W antibody recognized a conformational, discontinuous or - less likely - continuous epitope, because deletions of parts of the Ig-fold are likely to disrupt the native folding of the polypeptide. This conformation-dependency, however, also suggested that the lamin Ig-fold had refolded, at least partially, on Western blot membranes after SDS-PAGE.

### Specific detection of lamin A/C mutant proteins in primary patient skin fibroblasts

Since most laminopathy-associated *LMNA* mutations are inherited as dominant traits, point-mutant specific antibodies would allow for the first time to study in patient cells potential alterations of the mutant protein, such as sub-cellular localization, protein interactions, or post-translational modifications, a task, for which so far no research tools have been available that could distinguish the point-mutant from the wild-type protein. Hence, we next examined whether the unique features of our novel antibodies could also be employed to investigate by Western blotting, immunofluorescence staining, and immunoprecipitation the mutant forms of lamin A and lamin C in diseased primary human cells. For these studies, skin fibroblasts from an EDMD patient heterozygous for the R453W *LMNA* mutation, as well as skin fibroblasts from an FPLD patient heterozygous for the R482W *LMNA* mutation were used.

**Figure 5:**
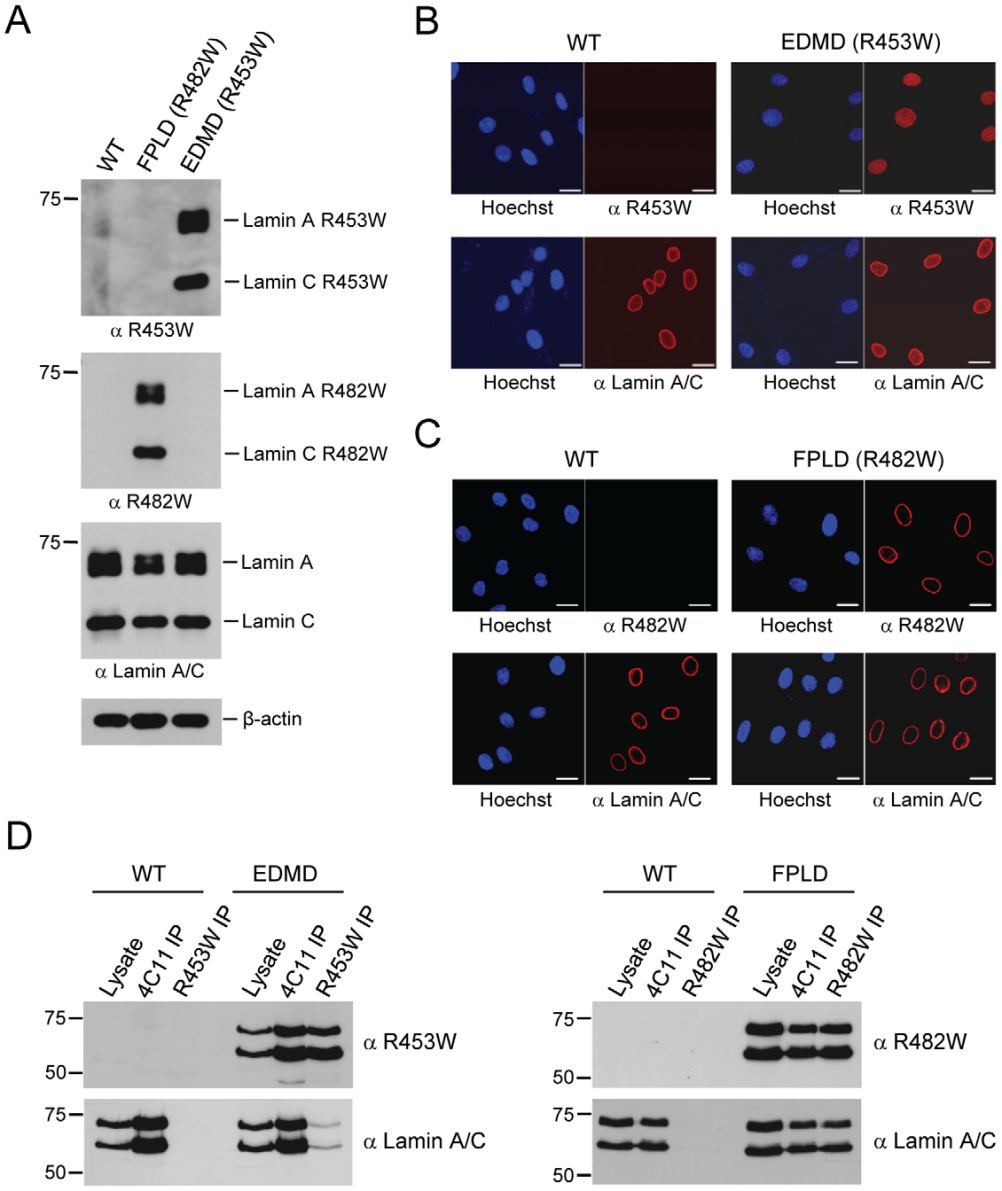
R453W and R482W antibodies recognize the respective disease causing mutant A-type lamins in primary patient fibroblasts. (**A**) Western blot analysis of wild type, EDMD (R453W), and FPLD (R482W) primary human fibroblast lysates with anti R453W (panel I), anti R482W (panel II), anti Lamin A/C 4C11 (panel III), and anti β-actin (panel IV). (**B**) Immunofluorescence of wild-type and EDMD primary human fibroblasts. Cells were stained with anti R453W (top panels) and anti Lamin A/C 4C11 (bottom panels). The DNA was counterstained with Hoechst 33342. Bar scale, 20 µm. (**C**) Immunofluorescence of wild-type and FPLD primary human fibroblasts. Cells were stained with anti R482W (top panels) and anti Lamin A/C 4C11 (bottom panels). The DNA was counterstained with Hoechst 33342. Bar scale, 20 µm. (**D**) Immunoprecipitation of wild-type and mutant lamin A/C from wild-type, EDMD, and FPLD cell lysates. The precipitated proteins were separated by 7.5% SDS-PAGE and analyzed by Western blotting with either the anti R453W or anti R482W antibody (top panels), or with anti Lamin A/C 4C11 (bottom panels).

The R453W antibody detected lamin A and lamin C only in the EDMD fibroblast lysate, whereas the R482W antibody recognized lamin A and lamin C only in the lysate of FPLD fibroblasts (Figure 5A, panel I and II). Similar to the results obtained in the HeLa cell system, neither antibody cross-reacted with the other mutant or detected the wild-type proteins in control skin fibroblasts from a healthy donor.

Furthermore, both antibodies also displayed unique specificity for the respective lamin A/C mutants by immunofluorescence. The general lamin A/C antibody stained the nuclei in wild-type control as well as EDMD and FPLD cells (Figure 5B+C, lower panels). In contrast, cell staining with the mutant-specific antibodies was only observed in the EDMD or the FPLD cells, respectively (Figure 5B+C, upper panels), underlining the specificity of these antibodies. Interestingly, the staining with the R453W antibody in EDMD fibroblasts indicated that a fraction of the lamin A/C R453W mutant might be partially aggregated in the nucleoplasm, while the R482W antibody primarily stained the nuclear envelope in FPLD fibroblasts. In agreement with earlier reports [33], [34], we observed a high percentage of FPLD patient cells containing grossly mis-shaped nuclei with invaginations and/or blebs, which were also apparent by staining with general lamin A/C antibodies (data not shown) or Hoechst DNA staining (Figure S2A). In contrast to one of these reports [33], however, we did not detect any significant differences in prelamin A - or vice versa in mature lamin A - levels between FPLD, wild-type or EDMD cells (Figure S2B).

Finally, we also tested the potential of the two novel antibodies to immunoprecipitate the respective mutant proteins. Indeed, both antibodies specifically immunoprecipitated the mutant proteins from the respective patient cells, but did not pull down any lamin A/C from wild-type cells (Figure 5D, top panels), again highlighting the specificity of both antibodies.

### The wild-type and R453W mutant protein localize differently in EDMD fibroblasts

**Figure 6:**
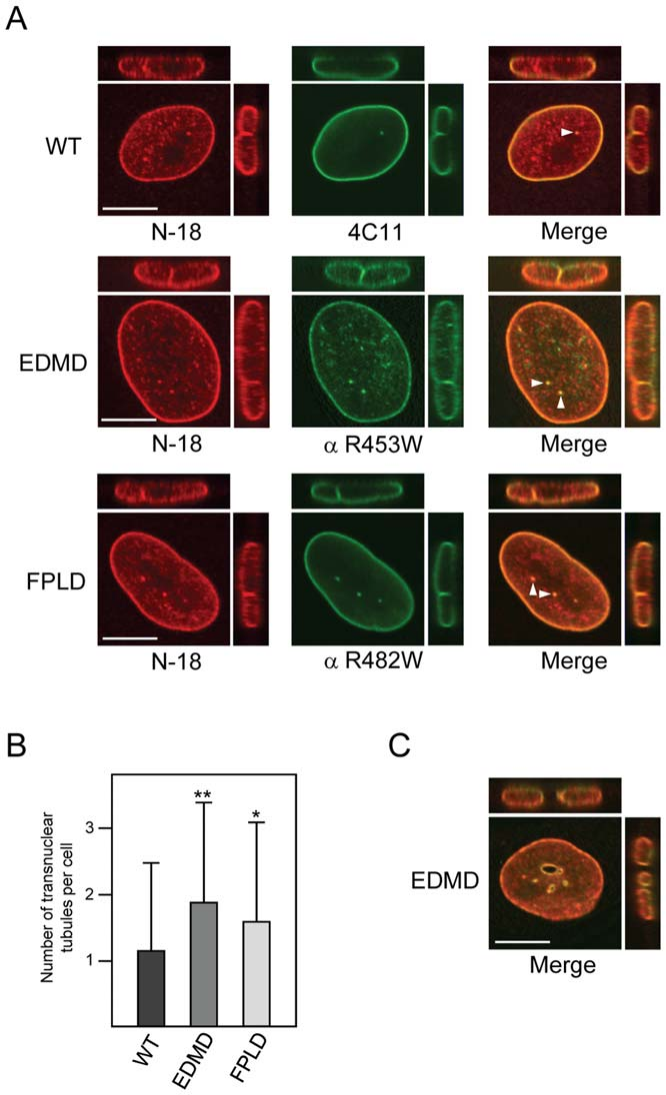
EDMD patient cells contain an increased number of transnuclear tubules. (**A**) Immunofluorescence of wild-type (top panel), EDMD (middle panel), and FPLD (bottom panel) primary human fibroblasts. Cells were co-stained with anti Lamin A/C N-18 and anti Lamin A/C 4C11, anti R453W, or anti R482W, respectively. Mid plane sections, xz-projections (above), and yz-projections (right) of the nuclei are shown. Arrowheads indicate transnuclear tubules through which the respective projection planes cut. Bar scale, 10 µm. (**B**) Number of transnuclear tubules per cell for wild-type, EDMD, and FPLD fibroblasts. n = 44 (wild-type), n = 46 (EDMD), n = 50 (FPLD). ** p<0.02; * p = 0.13. (**C**) Merged image of a mid plane section, xz-projection (above), and yz-projection (right) of an EDMD nucleus. Bar scale, 10 µm.

To further investigate in the primary human wild-type or patient fibroblasts the subnuclear distribution of A-type lamins we performed double cell stainings with a widely used goat polyclonal antibody recognizing a Lamin A/C N-terminal epitope, N-18 (Santa Cruz) [35], [36], [37], in combination with 4C11, anti R453W, or anti R482W (Figure 6A). While antibody 4C11 predominantly stained the nuclear envelope in all cell types (Figure 6A, upper panel and data not shown), antibody N-18 also recognized lamin A/C in the nucleoplasm. The double stainings revealed very prominent co-localizing foci in the nuclear interior of all three cell types (Figure 6A, arrowheads). Serial optical sections along the z axis showed, that these foci represented continuous structures either ranging from one side of the nucleus to the other, or penetrating from the nuclear envelope deep into the nuclear interior (Figure 6, upright projections). Similar tubular structures have been shown to consist of two membranes continuous with the nuclear envelope and to be decorated on the nucleoplasmic side with lamins [38]. Interestingly, EDMD patient cells contained a significantly higher number of transnuclear tubules than the wild-type cells (Figure 6B). While we also observed an increased number of transnuclear tubules in FPLD cells, the difference to wild-type cells was statistically not significant. In rare cases, we even observed very wide channels with diameters of several µm in EDMD cells (Figure 6C), suggesting that nuclear reassembly might be affected in these cells [38].

**Figure 7:**
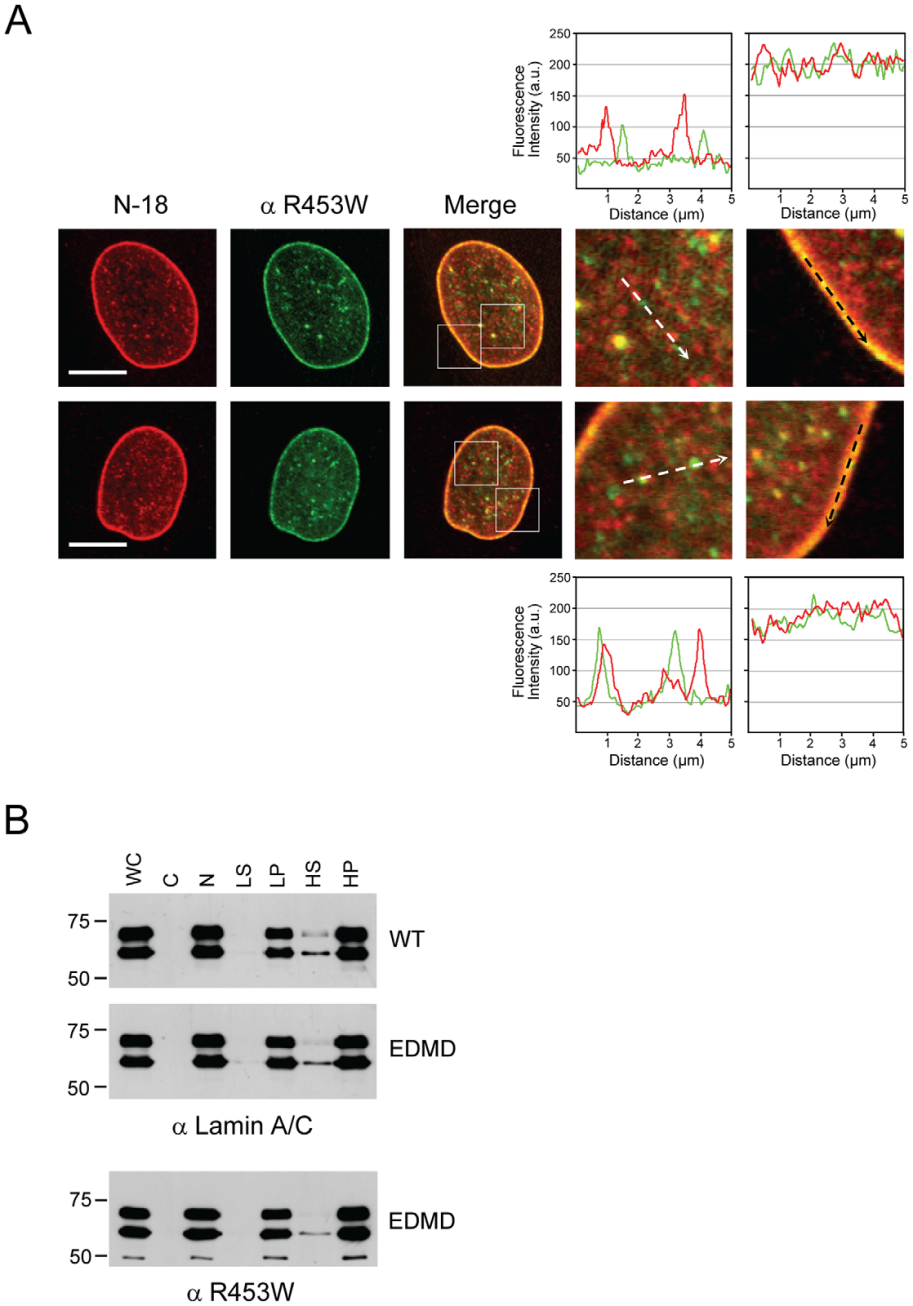
Wild-type and mutant R453W lamin A/C are present in distinct, non co-localizing foci in EDMD nuclei. (**A**) Immunofluorescence of two EDMD nuclei. Cells were co-stained with anti Lamin A/C N-18 and anti R453W. Fluorescence intensity of the lamin A/C staining by the two antibodies along the dashed lines in the magnified images of the nucleoplasm (4^th^ image in each row) and of the nuclear rim (last image in each row) are shown above or below the respective images. Areas of magnification (3.5-fold) are indicated by white squares in the merged images of each nucleus. Bar scale, 10 µm. (**B**) Cellular fractionation of wild-type and EDMD cells. Whole cell lysate (WC), cytoplasmic (C), whole nuclear (N), low-salt soluble nuclear (LS), low-salt insoluble nuclear (LP), high-salt soluble nuclear (HS), and high-salt insoluble nuclear (HP) fractions were separated by 7.5% SDS-PAGE and analyzed by Western blotting with the indicated antibodies.

Close inspection of the intranuclear N-18- or R453W-specific stainings in horizontal mid-plane sections of EDMD nuclei revealed well-defined lamin foci of varying size (Figure 7A). Several of these foci were co-stained by both antibodies and represented mostly either transnuclear tubules or deep, narrow invaginations of the nuclear envelope. However, the majority of nucleoplasmic foci stained by the N-18 antibody did not colocalize with those stained by the R453W mutant-specific antibody (Figure 7A, enlarged details of nuclear interior). This finding suggested a distinct intranuclear distribution of the R453W mutant and the wild-type protein that could only be detected by the novel R453W mutant-specific antibody but not the N-18 polyclonal antibody, which was hampered in the detection of the intranuclear R453W mutant proteins. Finally, biochemical subcellular fractionation experiments, testing for salt/detergent resistant versus extractable lamin fractions, did not show any significant differences in the solubility of lamin A and C in the wild-type and EDMD primary cells (Figure 7B).

## Discussion

Here we present two novel monoclonal antibodies specifically detecting disease causing point mutants of A-type lamins, namely the AD-EDMD associated lamin A/C R453W mutant and the FPLD associated lamin A/C R482W mutant. While the R453W specific antibody was raised against a short peptide, we were unsuccessful in generating an R482W mutant specific antibody by a similar approach. Rather, immunization with a fusion protein consisting of the hepatitis B virus capsid protein, HBcAg, and the lamin A/C Ig-fold R482W domain inserted at the highly immunogenic viral c/e1 epitope resulted in the generation of the R482W specific antibody. In addition, by the latter approach using a HBcAg-Ig-fold wt fusion protein the 4C11 antibody was generated, which recognizes an as yet undefined epitope within the Ig-fold domain and detects lamin A/C wt, R453W, and R482W. Because of its excellent features in Western blotting, immunofluorescence, and immunoprecipiation, this antibody was used throughout this study to detect endogenous A-type lamins.

Interestingly, immunization with the HBcAg-Ig-fold R453W fusion protein did not result in the generation of a mutant-specific immune response (data not shown). AD-EDMD associated missense mutations in the *LMNA* gene are thought to result in misfolding of the protein or the failure to assemble into lamin A polymers [39], and Krimm et al. have shown that the substitution of arginine 453 by tryptophan causes a dramatic reduction of the denaturation temperature of the Ig-fold domain due to the loss of a salt bridge between R453 and E443/E444 [11]. Indeed, we observed a reduced solubility of the R453W mutant fusion protein compared to the HBcAg Ig-fold wt and R482W, suggesting improper folding of the recombinant protein due to the instability of the R453W mutant Ig-fold. We also note, that lamin A/C R453W could only be immunoprecipitated with the R453W specific antibody in the presence of 0.25% SDS (Figure 5D), supporting the idea of a destabilized structure of the mutant. Such a treatment most likely results in a partial denaturation of the mutant lamin A/C, thereby possibly making the epitope encompassing W453 accessible for the anti-peptide R453W antibody. However, fractionation assays of EDMD cells did not reveal significant differences in the solubility of the wild-type and the mutant lamin A/C proteins.

In the case of the R482W lamin A/C mutant, we were unable to obtain an R482W mutant specific immune response against the full-length protein when short peptides were used for immunizations (data not shown). However, when we used the entire Ig-fold domain harboring the R482W mutation for the immunizations, we readily obtained a R482W mutant specific immune response and could generate a R482W monoclonal antibody suggesting that the antigenicity of the R482W epitope depended on the conformational context of the Ig-fold that evidently could not be mimicked by a short linear peptide. Consistent with this, peptides (either 9 or 21 amino acids long) were neither detected by the R482W antibody nor could they impair binding of the antibody to Flag-lamin A R482W, indicating that, while W482 must be part of the functional epitope, the antibody was most likely recognizing a discontinuous epitope in a conformation dependent manner. Recently, the structure of the lamin A/C R482W mutant has been shown to be almost identical to that of the wild-type protein [40]. Thus, the R482W antibody does not differentiate between the wild-type and the mutant protein due to differences in their conformation, but rather specifically recognizes the amino acid side chain of W482, which has been shown to be solvent exposed on the protein surface.

Recently, several groups have reported the generation of specific antibodies against disease relevant point mutant proteins, namely glial fibrillary acidic protein R416W, epidermal growth factor receptor L858R, and isocitrate dehydrogenase R132H, respectively [41], [42], [43], [44]. In these studies, antibodies were raised against short peptides, which combine the advantages of easy and fast fabrication with the potential to design highly target-oriented antigens. However, most, if not all, antibodies recognize discontinuous epitopes, of which linear peptides represent only parts of the binding interface between antigen and antibody [31]. In perfect agreement with this notion, our findings show that - as for any protein - synthetic peptides representing short stretches of mutant proteins may be of very limited use, if a mutation affects a residue, which even if surface exposed is located at a structural position within the protein, which does not allow to be remodeled by a short linear amino acid chain that displays random structures not present in the full length protein [45]. As an alternative approach, the display of such residues in the context of a complete protein or a defined protein domain may lead to the desired immune response. Furthermore, even though we failed to obtain a mutant-specific immune response for the HBcAg Ig-fold R453W fusion protein, it is conceivable that for other proteins, where a certain mutation leads to gross but stable alterations in the three-dimensional structure of the protein, the immunization with the respective mutant protein or protein domain could also be beneficial for obtaining antibodies specific for the mutant conformation but not necessarily the mutated amino acid.

Several data presented here are in agreement with the idea that the R482W antibody binds specifically to lamin A/C R482W, yet only in a conformation-dependent manner when the whole Ig-fold domain is present (Figure 3B and Figure 4). However, since this antibody also detected specifically the mutant protein in the immunoblot analysis, these findings would suggest that after denaturing SDS-PAGE some proteins or protein domains can refold into their native conformation, thereby contradicting the general view of proteins being captured in a denatured state on a Western blotting membrane. It will be interesting to see whether this is a lamin A/C specific property, possibly because the Ig-fold adapts an unusually stable conformation. Indeed, the CD analysis of the Ig-fold reveals a melting temperature of 62°C, which is well above the melting temperature of most proteins and indicates a very stable structure [11]. Experiments are currently under way to further investigate this phenomenon.

The antibodies presented and characterized in this study open up completely new avenues to investigate the molecular mechanisms of two laminopathies, AD-EDMD and FPLD, in patient cells and tissues. So far the properties of disease-causing mutant A-type lamins were studied by the ectopic expression of GFP- or epitope-tagged proteins in the context of a variety of lamin A/C wild type cells, leading to conflicting results about a possible mislocalization of the two lamin A mutants [22], [34], [46], [47], [48]. By transient transfection of C2C12 cells with Flag-tagged lamin A mutants, Östlund et al. found that a sub-set of mutants, including several AD-EDMD-linked mutants, was dramatically mislocalized in many cells, but that all AD-EDMD mutants containing a mutation within the Ig-fold, and in particular the R453W mutant, showed no localization abnormalities. Likewise, localization of the R482W mutant was essentially indistinguishable from that of wild-type lamin A [22]. In contrast, Broers and colleagues found differences in the sub-nuclear localization of both, the R453W and the R482W lamin A and lamin C mutants compared to the wild type proteins in CHO-K1 cells stably transfected with GFP-lamin A or C fusion constructs [47]. Using GFP-tagged lamin, Wiesel et al. have also shown recently that the *C. elegans* equivalent of the human R453W mutant, Ce-lamin R460W, accumulates in nuclear aggregates [48]. While these studies revealed several interesting features of mutant Ce-lamins that may be similar to human lamin A mutants, and some of the cellular phenotypes resembled those seen in some patient cells [34], these results may not provide the whole picture. For instance, Ce-lamin is a B-type lamin and the only lamin expressed in worms, while mutant human lamins are A-type lamins and their localization may be affected by human B-type lamins and by lamin A/C binding proteins missing in *C. elegans*. Also, the fact, that in some studies immortalized rodent cell lines were used for the ectopic expression of mutant lamins, may have impacted on some of their disease-relevant properties. For instance, while most laminopathies are inherited as dominant traits in humans, pathological phenotypes due to amino acid substitutions become apparent in mouse models only under homozygous conditions, thereby highlighting considerable differences between humans and mice [21]. Furthermore, even though the localization of wild-type lamin A/C fused to GFP appeared normal, the GFP-tag might influence lamin properties and thus laminopathy-associated functions of mutant lamins. Finally, by changing the overall amount of A-type lamins by the additional expression of a tagged lamin A/C protein, mutant or wild-type, may also have an impact on lamin A/C properties in a cell. In a first attempt to investigate the properties of mutant lamins in primary patient cells using our novel antibodies, we examined their subnuclear distribution by confocal immunofluorescence microscopy. In EDMD fibroblasts, we found distinct lamin A/C foci in the nuclear interior that were detected either by the R453W-specific antibody or a general lamin A/C antibody. In contrast, the lamin A/C and R453W stainings showed near perfect colocalization at the nuclear envelope. It is therefore conceivable that antibody N-18 did not detect a sub-set of R453W-specific intranuclear foci because the epitope of the N-18 antibody in the lamin A/C N-terminus may be inaccessible in these structures. Hence, the R453W mutant lamin seems to exist in at least two different protein pools: one pool at the nuclear lamina and in transnuclear tubular extensions of the nuclear envelope, which is also detectable by antibody N-18; and a second pool in nucleoplasmic foci not co-stained by antibody N-18. The specific consequences of this unique R453W-specific pool remain unclear. It has been shown recently, that A- and B-type lamins form separate but interconnected meshworks and microdomains, which interact with different types of chromatin [37]. Thus, one could speculate that mutant lamin aggregates might affect chromatin organization. Interestingly, altered epigenetic chromatin modifications have been found in EDMD mouse models as well as patient muscles [49]. The two antibodies described here represent a starting point to study not only potential abnormalities in the sub-nuclear localization of lamin A/C R453W and R482W, but their excellent features in immunoblotting and immunoprecipitation assays will also allow to study changes in the post-translational modification status, protein stability and expression levels, as well as the interacting proteome of the mutant proteins. We propose that the generation of similar mutant-specific antibodies will provide a way to study new aspects of many hereditary or acquired somatic diseases in primary human cells, which up to now have escaped direct experimental analysis.

## Supporting Information

Figure S1 Anti lamin A/C, clone 4C11, recognizes wt, R453W, and R482W Ig-folds. 12.5% SDS-PAGE/Western blot analysis of Ni-agarose purified recombinant His-tagged HBcAg fusion proteins with anti His (left panel) or anti lamin A/C, clone 4C11 (right panel).
(0.19 MB TIF)

Figure S2 Increased number of dysmorphic nuclei in FPLD fibroblasts. (A) Representative examples of dysmorphic nuclei found in FPLD cells. Cells were stained with anti R482W and DNA was counterstained with Hoechst 33342. Bar scale, 10 µm. The percentage of dysmorphic nuclei for wild-type (p36), EDMD (p34), and FPLD (p32) cells is displayed in the diagram. n = 100 for each cell type (B) SDS-PAGE/Western blot analysis of wild-type, EDMD, and FPLD fibroblast lysates at the same passage numbers as in (A) with anti Prelamin A (top panel), anti mature Lamin A 4A4 (middle panel), and anti Lamin A/C 4C11. A lysate of 16 hours FTI-277 treated T98G cells served as a positive control for Prelamin A.
(0.34 MB TIF)
