# Supplementary figures and images for: Monoclonal Antibodies Specific for Disease-Associated Point-Mutants: Lamin A/C R453W and R482W
Source: PLoS One. 2010 May 13;5(5):e10604. doi: 10.1371/journal.pone.0010604 (PMC2869350; doi:10.1371/journal.pone.0010604)

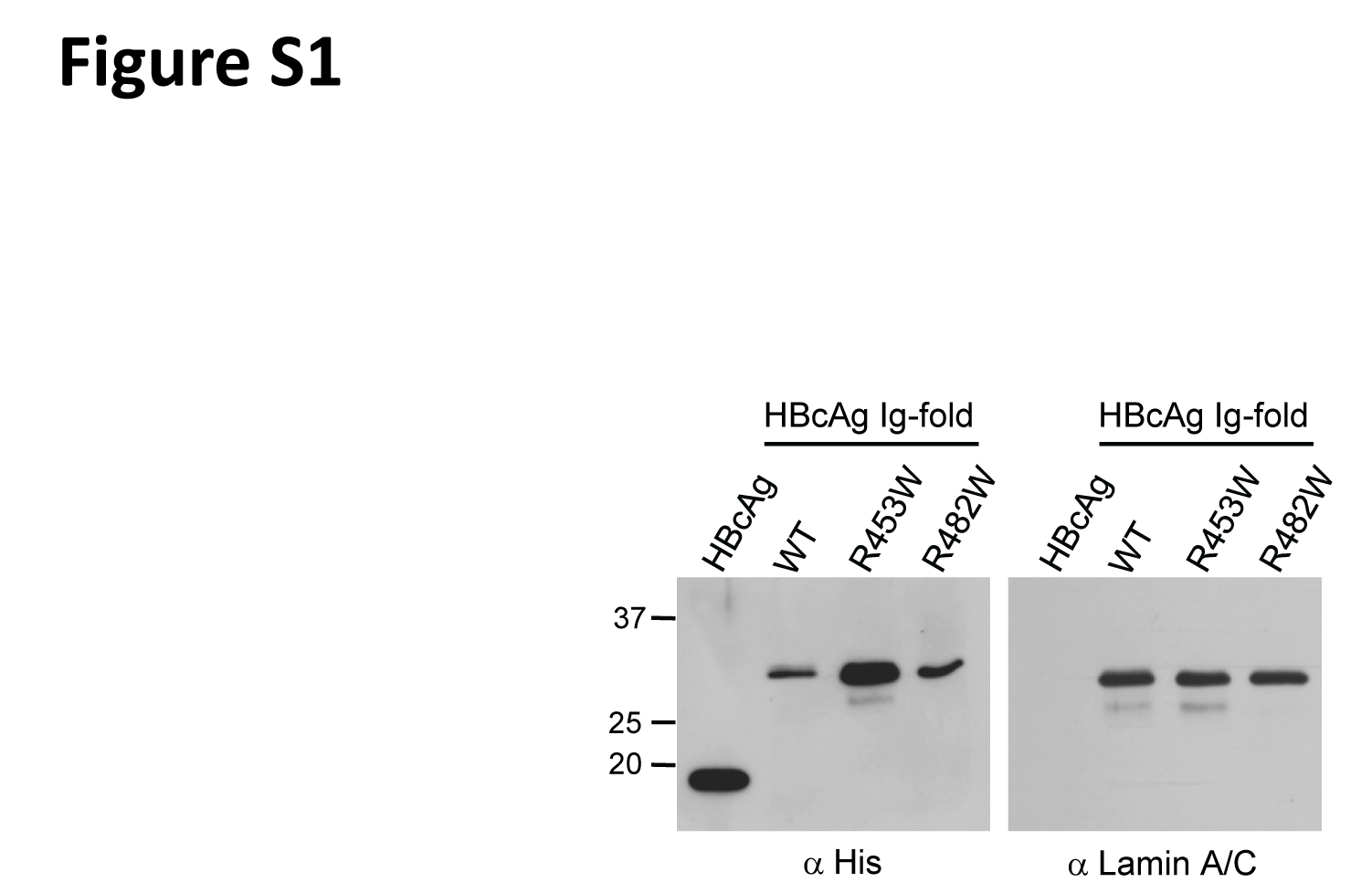

Supplement: Figure S1 — Anti lamin A/C, clone 4C11, recognizes wt, R453W, and R482W Ig-folds. 12.5% SDS-PAGE/Western blot analysis of Ni-agarose purified recombinant His-tagged HBcAg fusion proteins with anti His (left panel) or anti lamin A/C, clone 4C11 (right panel). (0.19 MB TIF) [file pone.0010604.s001.tif]

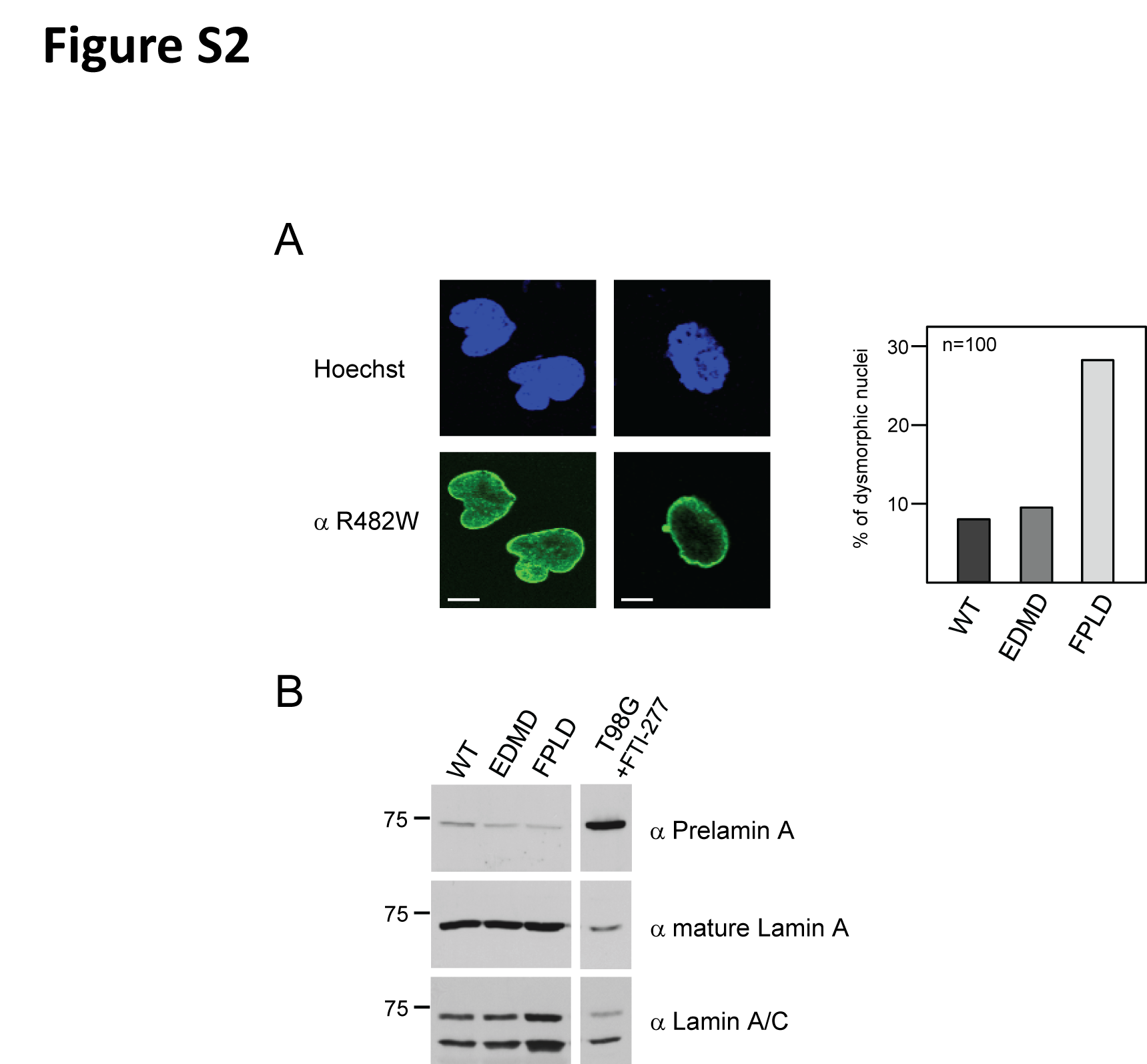

Supplement: Figure S2 — Increased number of dysmorphic nuclei in FPLD fibroblasts. (A) Representative examples of dysmorphic nuclei found in FPLD cells. Cells were stained with anti R482W and DNA was counterstained with Hoechst 33342. Bar scale, 10 µm. The percentage of dysmorphic nuclei for wild-type (p36), EDMD (p34), and FPLD (p32) cells is displayed in the diagram. n = 100 for each cell type (B) SDS-PAGE/Western blot analysis of wild-type, EDMD, and FPLD fibroblast lysates at the same passage numbers as in (A) with anti Prelamin A (top panel), anti mature Lamin A 4A4 (middle panel), and anti Lamin A/C 4C11. A lysate of 16 hours FTI-277 treated T98G cells served as a positive control for Prelamin A. (0.34 MB TIF) [file pone.0010604.s002.tif]
